# Clear Cell Carcinoma of the Endometrium: Evaluation of Prognostic Parameters in 27 Cases

**DOI:** 10.3389/fonc.2021.732782

**Published:** 2021-12-02

**Authors:** Zhiyang Zhang, Penglian Gao, Zhengqi Bao, Linggong Zeng, Junyi Yao, Damin Chai, Tian Li

**Affiliations:** ^1^ Department of Pathology, The First Affiliated Hospital of Bengbu Medical University, Bengbu, China; ^2^ Department of Orthopedics, The First Affiliated Hospital of Bengbu Medical University, Bengbu, China; ^3^ School of Basic Medicine, Fourth Military Medical University, Xi’an, China

**Keywords:** clear cell endometrial carcinoma, clinicopathology, prognosis, overall survival, clinical study

## Abstract

**Objective:**

Clear cell carcinoma (CCC) of the endometrium is an uncommon yet aggressive tumor. Few cohort studies are reporting the overall survival time of CCC patients. This study aimed to retrospectively analyze the clinicopathologic features, molecular characteristics and survival data of 27 endometrial CCC patients to improve the understanding of CCC.

**Methods:**

The clinicopathologic features, molecular characteristics and survival data total of 27 CCC patients admitted to the BBMU affiliated hospital (Anhui, China) between January 2005 and December 2018 were retrospectively analyzed. Kaplan-Meier method was used to analyze the prognosis-related factors.

**Results:**

The median age of the patients was 60 years (range; 39 to 81 years). The average tumor size was 3.8 cm (range; 0.8 to 13.0cm). Myometrial infiltration greater than 50% was reported in 55.6% of the patients, while the Ki-67 index greater than 50% was reported in 70.4% of the patients. The patients’ FIGO (2009) surgical stages were as follows: 18 I, 3 II, 4 III, and 2 IV. Besides, 7 (25.6%) patients had lymphovascular invasion, 3 (11.1%) patients with distant metastasis, including 1 patient with bone metastasis, and 2 with liver metastasis. Adjuvant treatment included 7 with chemotherapy alone, 9 with radiotherapy alone, and 9 with both radiotherapy and chemotherapy. The median overall survival time from the time of CCC diagnosis was 56 months. ER and PR showed negative expression and P16 showed patchy immunostaining. 18 (63%) cases showed Napsin A positive expression. Loss of MSH2, MSH6 and PTEN were seen in 5, 4 and 7 cases respectively. All cases showed HER-2/nue negative expression.

**Conclusion:**

CCC is a rare and invasive tumor. Age of diagnosis, FIGO stage, tumor size, myometrial infiltration, lymphovascular invasion, distant metastasis, Ki-67 index and P53 expression are important indicators to evaluate patient’s prognosis (P = 0.048, P < 0.001, P = 0.016, P = 0.043, P = 0.001, P < 0.001, P = 0.026, and P = 0.007, respectively). CCC has a worse prognosis than endometrioid carcinoma (P = 0.002), and there is no significant difference when compared with uterine papillary serous carcinoma (P = 0.155).

## Introduction

Clear cell carcinoma (CCC) of the endometrium is accounting for 1%–5.5% of all endometrial carcinomas (1–3). Due to the lack of exploratory research, the incidence of CCC may be underestimated. Most CCC are a mixture of at least two architectural forms, the most common form is papillary and solid mixed growth (4). Compared with endometrioid carcinoma (EC), patients with CCC have a worse prognosis (5). The 5-year overall survival rate of patients with higher International Federation of Gynecology and Obstetrics (FIGO) stage is below 50% (6).

CCC can occur in different tissues, such as the vagina, uterus, cervix, and ovary (7). Due to the low incidence rate of CCC of the endometrium, data on prognosis-related factors of CCC is still controversial. Therefore, in this study, we retrospectively analyzed the clinicopathological parameters, immunohistochemical analysis results and survival data of 27 CCC patients diagnosed by immunohistochemistry to identify significant prognostic parameters.

## Materials and Methods

### Ethics Statement and Tissue Sample Collection

Tissue sample collection was approved by the Ethics Committee of the First Affiliated Hospital of Bengbu Medical College and informed consent was obtained from all the patients. A retrospective review of endometrial cancer cases diagnosed from January 2005 to December 2018 was conducted. A total of 222 cases with a diagnosis of endometrial cancer were included, 27 cases with CCC, 45 cases with uterine papillary serous carcinoma (UPSC) and 150 cases with EC, and those cases without histological and cytological confirmation were excluded. Prognostic parameters including, age, tumor size, FIGO stage, myometrial invasion (MI), lymphovascular invasion (LVI), distant metastasis, adjuvant therapy was reviewed to determine vital status and overall survival (OS). Depth of MI was measured from the deepest part of the lesion to the serosa with the naked eye. An expert group comprising of three experienced pathologists who were blinded to the patients’ clinical results, re-evaluated the pathological sections according to the World Health Organization (WHO) criteria of CCC.

### Immunohistochemical Analysis and Interpretation

A total of 222 specimens were processed using classical histological methods, including 10% buffered formalin fixation, paraffin embedding, HE staining, and immunohistochemical staining. Immunohistochemical analysis was performed using the ElivisionTM Plus detection kit (Lab Vision, USA) according to the manufacturer’s instructions to determine the expression of estrogen receptors (ER), progesterone receptors (PR), P53 protein, Napsin A, and Ki-67. In addition, we performed the immunohistochemical analysis of mismatch repair (MMR) proteins (MSH2, MSH6), p16 protein, PTEN, and HER-2/neu in 27 CCC cases. The antibodies were purchased from Maixin Biotechnology Co., Ltd. (Fuzhou, China). Immunohistochemical staining can clearly distinguish CCC, UPSC and EC.

Upon immunohistochemical staining, the positive expression results showed brownish-yellow granule deposition, while cases without staining were considered negative. The Allred scoring system was used to evaluate ER and PR. Score according to the intensity and degree of staining. The intensity of staining score: 0 – no-staining, 1 – weak, 2 – moderate, 3 – strong. The extent of positive staining was graded as follows: 1, ≤ 1%; 2, 1% - 10%; 3, 10% - 33%; 4, 33% - 66% and 5, 66% - 100%. The score was interpreted as > 2 is positive expression (8). HER2/neu, P16, P53 and Napsin A expression was evaluated as described previously. If <10% of the tumor cells was stained considered as negative expression (9–11). ER, PR and P53 were nuclear expression. HER2/neu was located in membrane. Napsin A was expressed in the cytoplasm. P16 was presented as nucleus and cytoplasm. MMR proteins (MSH2 and MSH6) and PTEN were considered abnormal if loss of nuclear expression. Peripheral lymphocytes and normal endometrium were regarded as positive internal control. Ki-67 was presented as nuclear expression. Immunostaining for Ki-67 was defined as a high expression if > 50% of the tumor cells were stained, but if < 50% stained, this was considered as low expression of Ki-67.

### Follow-up

All patients were followed up by telephone calls. OS was defined as the date of surgery to the date of death or last follow-up. The follow-up was completed in December 2020.

### Statistical Analysis

SPSS 25.0 (IBM Corp., NY) statistical software was used to perform statistical analysis. Kaplan-Meier method was used to conduct univariate analysis to evaluate the relationship between clinicopathological data and survival rate. The Chi-square test was used for classified variables, while the independent sample t-test was used for continuous variables. P < 0.05 was considered to be statistically significant.

## 3 Result

### Clinicopathologic Features

A total of 27 patients diagnosed with CCC, and confirmed by immunohistochemical analysis of ER, PR, P16, Napsin A, P53 and Ki-67. The clinicopathological parameters of the included patients are summarized in **Table 1**. Typical clinical presentations included abnormal bleeding, pelvic pain, abdominal distension and pain. The most common clinical manifestation was postmenopausal bleeding or dysfunctional uterine bleeding. All the included patients were post-menopausal women except one. According to the FIGO staging system, the proportion of stage I was 18 cases (66.7%), there were 3 cases (11.1%) in stage II, and 6 (22.2%) patients in stage III and IV. Until December 2020, 9 (33.3%) patients died, with an average survival time of 18.4 months. The OS of patients with stage III and IV was significantly shorter than patients with stage I and II (median OS, 26 months compared with 67 months, P < 0.001).

**Table 1:** Clinicopathological characteristics in the 27 Patients included in this study.

Case	Age (year)	FIGO stage	MI	Size (cm)	LIV	Distant metastasis	Treatment	Status	OS (mo)
1	58	I a	<1/2	1.5	Absent	Absent	Surgery + TC	Alive	78
2	67	IV	>1/2	7.5	Present	Absent	Surgery + TC	Death	5
3	69	I b	>1/2	3.0	Absent	Absent	Surgery + AP + RT	Death	32
4	66	I a	<1/2	2.0	Absent	Absent	Surgery + RT	Alive	65
5	57	I a	Absent	1.0	Absent	Absent	Surgery	Alive	60
6	56	I a	Absent	2.5	Absent	Absent	Surgery	Alive	56
7	71	I a	<1/2	5.0	Absent	Absent	Surgery + RT	Alive	88
8	49	I b	>1/2	4.5	Absent	Absent	Surgery + AP + RT	Death	25
9	81	II	>1/2	3.5	Absent	Absent	Surgery + TC + RT	Alive	37
10	65	II	>1/2	4.0	Absent	Absent	Surgery + TC + RT	Alive	74
11	42	I b	>1/2	6.0	Absent	Absent	Surgery + AP + RT	Alive	45
12	72	I a	<1/2	2.5	Absent	Absent	Surgery + RT	Alive	67
13	39	I b	>1/2	4.0	Absent	Absent	Surgery +TC	Alive	100
14	65	III	>1/2	8.5	Present	Present	Surgery + AP	Death	26
15	63	IV	>1/2	6.5	Present	Present	Surgery + TC + RT	Death	8
16	49	III	>1/2	3.0	Present	Present	Surgery +TC + RT	Alive	76
17	58	I a	<1/2	3.0	Absent	Absent	Surgery + RT	Alive	96
18	50	I a	<1/2	1.5	Absent	Absent	Surgery + RT	Alive	128
19	54	I a	<1/2	1.0	Absent	Absent	Surgery + RT	Alive	87
20	57	I a	<1/2	2.5	Absent	Absent	Surgery + CAP	Death	29
21	64	I b	>1/2	13.0	Absent	Absent	Surgery + RT	Death	24
22	72	III	>1/2	5.0	Present	Present	Surgery + TC + RT	Death	5
23	75	I b	>1/2	2.5	Absent	Absent	Surgery + CAP	Alive	67
24	59	I a	Absent	0.8	Absent	Absent	Surgery + RT	Alive	118
25	52	III	>1/2	4.5	Present	Present	Surgery + TC + RT	Alive	38
26	64	I a	<1/2	2.0	Absent	Absent	Surgery + RT	Alive	74
27	65	II	>1/2	3.5	Present	Present	Surgery + TC	Death	12

MI, myometrial invasion; LVI, Lymphovascular invasion; OS, overall survival; RT, radiation therapy; CAP, cisplatin + cyclophosphamide + epirubicin; AP, cisplatin + epirubicin; TC, carboplatin + paclitaxel.

The average age at diagnosis was 60 years (range: 39 - to 81 years), 14 (52%) of the patients were older than 60 years, and the median age was 66 years. There were 14 cases with tumor diameter < 3.5cm and 13 cases with tumor diameter ≥ 3.5cm. The average tumor size was 3.5cm (range: 0.8cm to 13.0cm), and 14 (52%) of the tumors were more than 3.5cm. Myometrial invasion (MI) > 1/2 (extending to the outer half) was reported in 15 (55%) patients, while 9 (33%) patients had MI < 1/2 (inner half involvement), and 3 (11%) patients reported no invasion. Lymphovascular invasion was observed in 26% (7/27) of the cases, and distant metastases were reported in 3 (11.1%) patients.

### Morphological and Immunohistochemical Features

**Figure 1:**
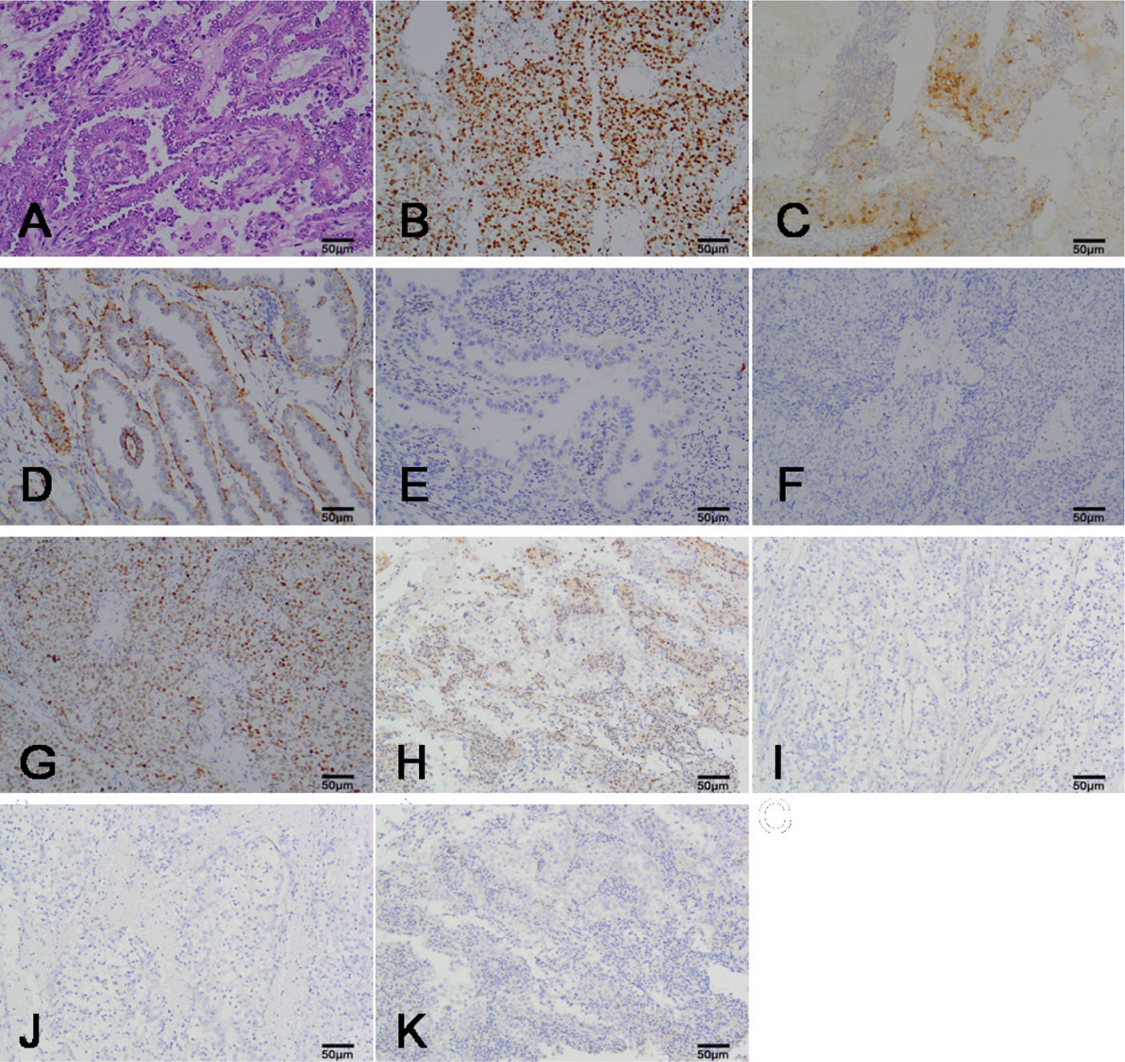
The representation micrographs showing of clear cell carcinoma of the endometrium (hematoxylin eosin stain for A, ×200; immumohistochemical stain, ×200 for B-K). **(A)** Papillary architecture and hobnail cells. **(B)** P16 staining. Nuclear and cytoplasm is patchy positive. **(C)** Ki-67 staining. Nuclear expression is positive. **(D)** Napsin A staining. Cytoplasm expression is positive. **(E)** ER staining. Nuclear expression is negative. **(F)** PR staining. Nuclear expression is negative. **(G)** P53 staining. Nuclear expression is positive. **(H)** PTEN staining. Nuclear expression is negative. Peripheral lymphocyte is positive. **(I)** MSH2 staining. Nuclear expression is negative. **(J)** MSH6 staining. Nuclear expression is negative. **(K)** HER-2/nue staining. Membrane expression is negative. Scar bar = 50μm. ER, estrogen receptors; PR, progesterone receptors.

The CCC of the endometrium can be observed in four morphological structures under a microscope, with the most common being papillary, followed by tubular cystic and solid structures. Cytoplasm clarity intermixed with eosinophilic cells and hobnail cells are the most prominent diagnostic feature of CCC. In this study, the majority of cases were mainly composed of hobnail cells (**Figure 1A**); a few cases intermixed with other cell types such as cubic cell and flat cell.

A total of 19 cases had a Ki-67 proliferation index higher than 50% (high expression), while 8 cases had a Ki-67 proliferation index lower than 50% (low expression). Patients with Ki-67 high expression had a shorter survival time compared with those with low Ki-67 proliferation indices (P = 0.026). All cases showed patchy immunostaining for P16. Positive expression of Napsin A was observed in 18 (63.0%) cases and Napsin A positive expression was unrelated to prognosis (P = 0.119). ER, PR is negative expression or focal expression in CCC. The immunohistochemical stain of P16, Ki-67, Napsin A, ER and PR in CCC was showed in **Figures 1B–F**.

In addition, we performed the immunohistochemical analysis of mismatch repair (MMR) proteins (MSH2, MSH6), P53, PTEN, and HER-2/neu to understand CCC from the perspective of molecular genetics. The immunohistochemistry of the 27 CCC patients are summarized in **Table 2**. Positive expression of P53 was observed in 17 (63.0%) cases and most of the cases were weakly or moderately positively expressed. Patients with positive expression of P53 had worse outcomes than those with negative expression of P53 (P = 0.007). Losses of MSH2 and MSH6 were seen in 5 and 4 cases, respectively. Loss of both MSH2 and MSH6 was observed in 7 cases (25.9%). PTEN loss was observed in 12 (44.4%) cases. Overexpression of HER2/neu was not found in all 27 cases. The immunohistochemical stain of P53, PTEN, MSH2, MSH2 and HER-2/neu in CCC was showed in **Figures 1G–K**.

**Table 2:** Immunohistochemistry of clear cell carcinoma of endometrium (n = 27).

Case	ER	PR	P16	Napsin A	P53	HER-2	PTEN	MSH2	MSH6	Ki-67(%)
1	–	–	+	–	–	–	–	+	+	20
2	–	–	+	+	+	–	+	+	+	60
3	–	–	+	–	+	–	+	+	+	85
4	–	–	+	–	–	–	–	–	–	70
5	–	–	+	+	+	–	–	+	+	20
6	–	–	+	+	+	–	+	+	+	60
7	–	–	+	+	–	–	+	+	+	70
8	–	–	+	+	+	–	–	+	–	70
9	–	–	+	+	+	–	–	+	+	80
10	–	–	+	+	–	–	+	+	+	80
11	–	–	+	+	+	–	–	+	+	20
12	–	–	+	+	–	–	–	–	+	20
13	–	–	+	+	–	–	+	+	+	90
14	–	–	+	–	+	–	+	–	–	80
15	–	–	+	+	+	–	–	+	+	90
16	–	–	+	+	+	–	+	+	+	30
17	–	–	+	+	+	–	+	–	+	80
18	–	–	+	+	+	–	–	–	+	80
19	+	–	+	–	–	–	+	+	+	30
20	–	–	+	–	+	–	+	+	+	70
21	–	–	+	+	+	–	+	+	+	90
22	–	–	+	–	+	–	+	+	+	90
23	–	–	+	+	–	–	–	+	+	80
24	–	–	+	–	–	–	+	+	+	20
25	–	–	+	+	+	–	+	+	–	80
26	–	–	+	+	–	–	–	+	+	30
27	–	–	+	–	+	–	–	+	+	80

ER, estrogen receptors; PR, progesterone receptors.

### Imaging Features

**Figure 2:**
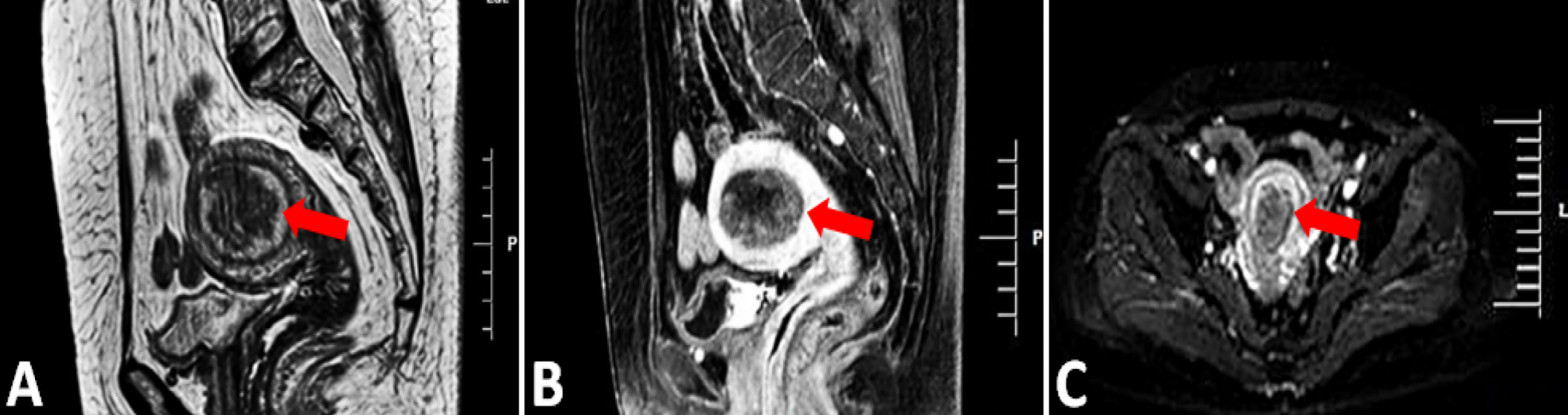
MRI, Magnetic resonance imaging. **(A, B)** Sagittal T2-weighted and T1 enhancement image showing the 5.2CM*3.5CM mass in the uterine cavity, the abnormal signal affected more than 1/2 of the myometrium (arrow). **(C)** Axial diffusion weighted image showing hyperintense endometrium (red arrow).

Preoperative pelvic magnetic resonance imaging of the uterus is shown in **Figures 2A–C**. Magnetic resonance imaging (MRI) showed hypointense or isointense on T1-weighted images, hyperintense on T2-weighted and T1 enhancement images and high signal intensity on diffusion weighted images. The normal postmenopausal endometrium is thin and homogeneous, the uterus with a thicken endometrium, irregular margins and irregular endometrial-myometrial border suggest endometrial carcinoma.

### Treatment and Outcomes

The cohort consisted of 27 patients, all patients were treated with abdominal hysterectomy (TAH) and bilateral salpingo-oophorectomy (BSO), and 22 (82%) patients underwent lymph node dissection. A total of 21 (78%) patients were stage I – II, 9 (42%) patients received radiation theory along, 5 (24%) patients were treated with chemotherapy along [cisplatin + cyclophosphamide + epirubicin (CAP), cisplatin + epirubicin (AP) and carboplatin + paclitaxel (TC). 5 (24%) patients received both radiation and chemotherapy. Besides, 2 (10%) patients received no adjuvant therapy. 6 (22%) patients were stage III - IV, 2 (33.3%) patients were treated with chemotherapy alone, and 4 (67%) patients received both radiation and chemotherapy. Chemotherapy was applied for at least six cycles. The sample size was small, therefore, it was impossible to determine if there was a significant difference between management and prognosis through subgroup analysis (P = 0.180).

In this study, three patients developed tumor recurrence: one developed bone metastasis following systemic treatment and died 3 months following recurrence; two patients developed liver metastasis after hysterectomy and died 5 months and 8 months after recurrence, respectively. No patient developed lung or brain metastasis. Patients with recurrent CCC were treated with systemic chemotherapy supplemented with external beam radiotherapy, and a chemotherapy regimen dominated by carboplatin and paclitaxel.

### Survival Analysis

A total of 27 patients were followed up until December 2020, and none of the patients was lost to follow-up. The patients had a median follow-up of 56 months (range: 5 - 128 months). During the follow-up interval, tumor-related deaths were observed in 9 (33.3%) of the patients, and the survival time was 5 – 32 months. The average survival time was 18.4 months, the median survival time was 24 months, and the 2-year OS rate was 81.5%.

**Figure 3:**
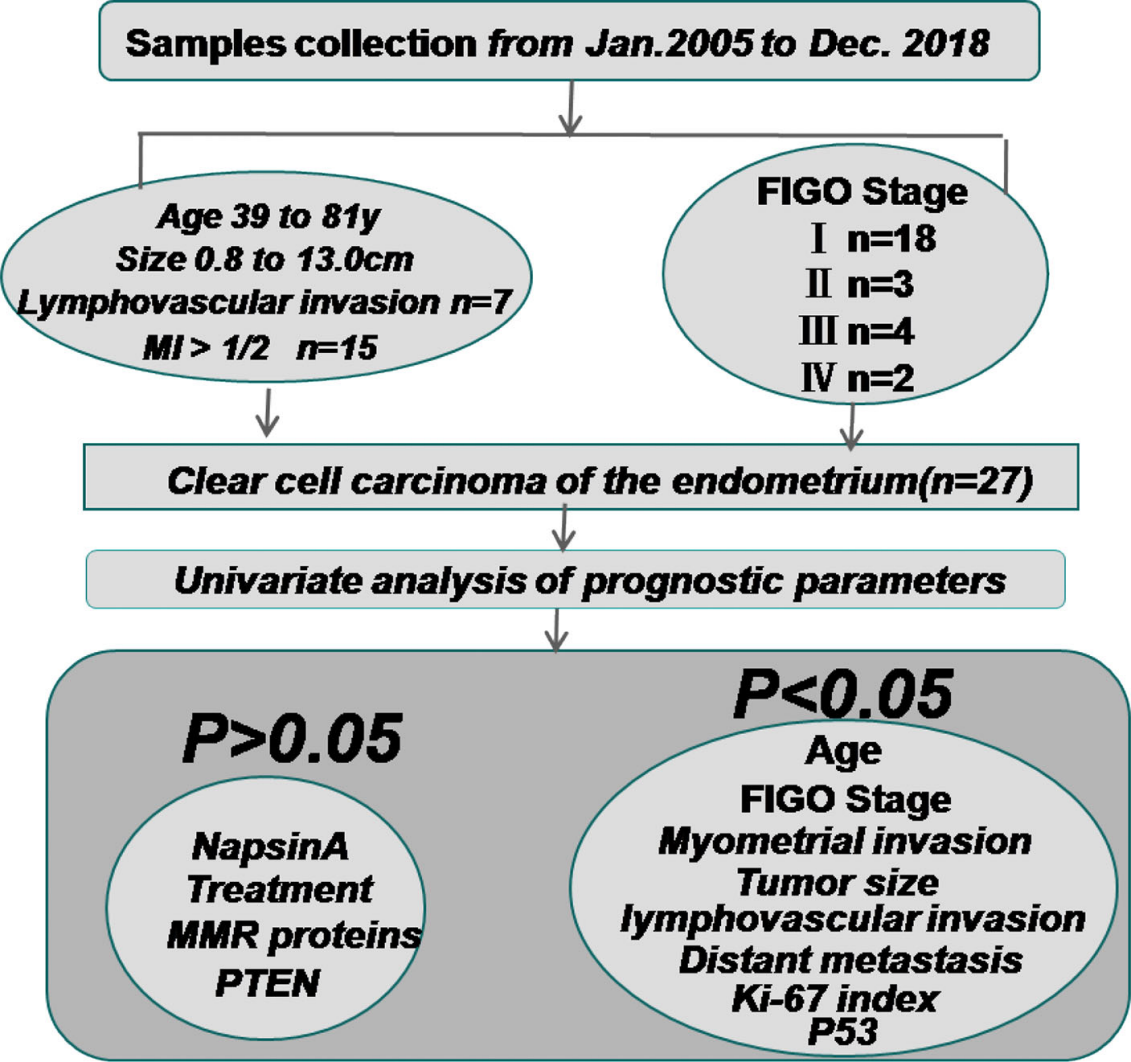
The clinicopathologic features and univariate analysis of prognostic parameters in clear cell carcinoma of the endometrium. Sample collection was approved by the Ethics Committee of the First Affiliated Hospital of Bengbu Medical College from January 2005 to December 2018. The patients’ FIGO (2009) surgical stages were as follows: 18 I, 3 II, 4 III, and 2 IV. The average age at diagnosis was 60 years (range: 39 - to 81 years). 7 patients had lymphovascular invasion and 15 patients had MI > 1/2. The Kaplan-Meier method was used for univariate analysis of the prognosis-related factors and OS. Age, FIGO stage, tumor size, MI, Ki-67 index, positive expression of P53, lymphovascular invasion, and distant metastasis of the patients were significantly associated with shorter OS ((P = 0.048, P < 0.001, P = 0.016, P = 0.043, P = 0.026, P = 0.007, P = 0.001, and P < 0.001 respectively). The positive expression of Napsin A (P = 0.119), adjuvant treatment (P = 0.180), loss of MSH2 (P = 0.472), MSH6 (P = 0.524) and PTEN (P = 0.472) were not statistically associated with OS. MI, myometrial invasion; MMR proteins, mismatch repair proteins.

**Figure 4:**
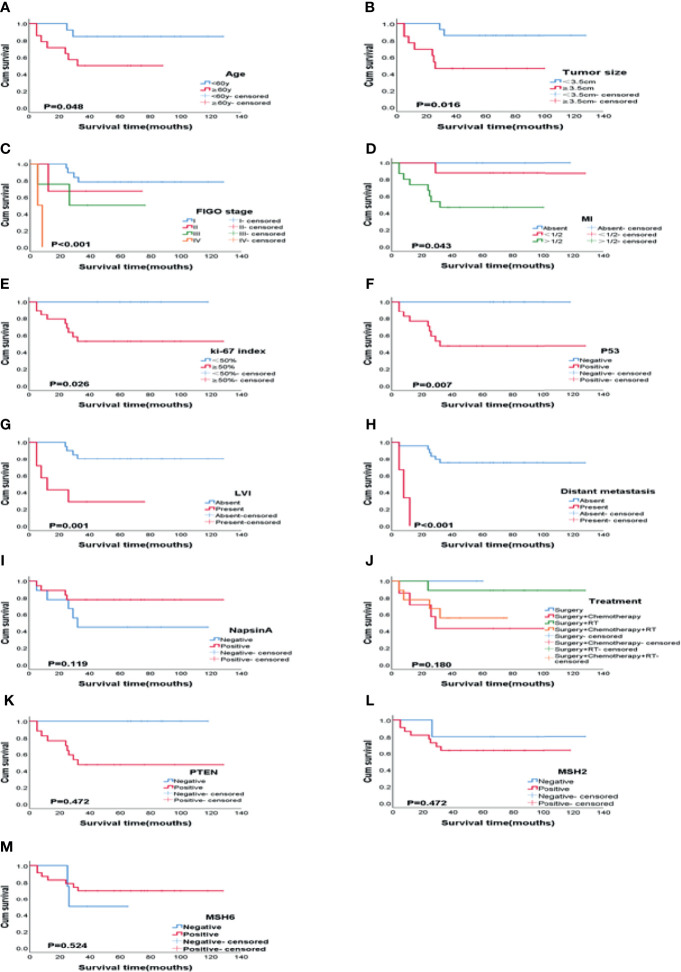
Kaplan-Meier plots of OS according to variables on univariate analysis. **(A)** Age (P = 0.048), **(B)** Tumor size (P = 0.016), **(C)** FIGO stage (P < 0.001), **(D)** MI (P = 0.043), **(E)** Ki-67 index (P = 0.026), **(F)** P53 (P = 0.007), **(G)** LVI (P = 0.001), **(H)** Distant metastasis (P < 0.001), **(I)** Napsin A (P = 0.119), **(J)** Treatment (P = 0.180), **(K)** PTEN (P = 0.472), **(L)** MSH2 (P = 0.472), **(M)** MSH6 (P = 0.524). MI, myometrial invasion; LVI, Lymphovascular invasion.

The Kaplan-Meier method was used for univariate analysis of the prognosis-related factors and OS of the patients. Older age, advanced FIGO stage (III - IV), big tumor size, deep MI, high Ki-67 index, positive expression of P53, lymphovascular invasion, and distant metastasis of the patients were significantly associated with shorter OS ((P = 0.048, P < 0.001, P = 0.016, P = 0.043, P = 0.026, P = 0.007, P = 0.001, and P < 0.001 respectively). The positive expression of Napsin A (P = 0.119), adjuvant treatment (P = 0.180), loss of MSH2 (P = 0.472), MSH6 (P = 0.524) and PTEN (P = 0.472) were not statistically significant in this study. **Figure 3** and **Table 3** shows the clinicopathologic features and univariate analysis of prognostic parameters in CCC, and the survival curves are shown in **Figures 4A–M**.

**Table 3:** Univariate analysis of overall survival (n = 27). .

Variable	n (%)	Log-Rank	P value
Age		3.916	**0.048**
<60y	13 (48.1)		
≥60y	14 (51.9)		
Tumor size		5.789	**0.016**
<3.5cm	14 (51.9)		
≥3.5cm	13 (48.1)		
Myometrial invasion		6.286	**0.043**
Absent	3 (11.1)		
<1/2	9 (33.3)		
>1/2	15 (55.6)		
Ki-67 index		4.945	**0.026**
<50%	8 (29.6)		
≥50%	19 (70.4)		
P53		7.174	**0.007**
Negative	10 (37.0)		
Positive	17 (63.0)		
Napsin A		2.430	0.119
Negative	9 (33.3)		
Positive	18 (66.7)		
PTEN		0.517	0.472
Negative	12 (44.4)		
Positive	15 (55.6)		
MSH2		0.518	0.472
Negative	5 (18.5)		
Positive	22 (81.5)		
LVI		10.157	**0.001**
Absent	20 (71.1)		
Present	7 (25.9)		
Distant metastasis		23.248	**<0.001**
Absent	24 (88.9)		
Present	3 (11.1)		
Treatment		4.890	0.180
Surgery	2 (7.4)		
Surgery + chemotherapy.	7 (25.9)		
Surgery + RT	9 (33.3)		
Surgery + chemotherapy + RT	9 (33.3)		
FIGO stage		20.749	**<0.001**
I	18 (66.7)		
II	3 (11.1)		
III	4 (14.8)		
IV	2 (7.4)		

Data in bold means statistically significant.

### Comparative Analysis of CCC, UPSC and EC

A total of 45 patients were diagnosed with uterine papillary serous carcinoma (UPSC), and the average age at diagnosis was 60 years (range: 44 - 76 years). A negative expression of ER was reported in 3 UPSC patients, while 38 patients showed P53 positive. Lymphovascular invasion was observed in 20 patients. There were 6 patients with distant metastasis, the common sites of metastasis were abdominal para-aortic lymph nodes, lung, liver and bone. Patients had an average survival time of 60 months. There was no difference in age, MI, treatment, LVI, distant metastasis, and OS between CCC and UPSC (P > 0.05). There was a significant difference in the expression of ER and P53 between CCC and UPSC (P < 0.001 and P = 0.038; **Table 4**). To detect ER/PR, P53 and Napsin A expression is important for the diagnosis and differentiation of UPSC and CCC. In USPC, P53 has a diffuse immunoreactivity.

**Table 4:** Comparative of clear cell carcinoma (CCC) of the endometrium, uterine papillary serous carcinoma (UPSC) and endometrioid carcinoma (EC).

Variable	CCC n = 27	PS n = 45	EC n = 150	P_1_ value CCC *vs* UPSC	P_2_ value CCC *vs* EC
Average age	60	60	56	0.467	0.380
(age range)	(39 - 81)	(44 - 76)	(31 - 79)		
Myometrial invasion				0.142	**0.039**
lt;1/2	12	28	98		
>1/2	15	17	52		
ER				**<0.001**	**<0.001**
Negative	26	3	12		
Positive	1	42	138		
PR				**<0.001**	**<0.001**
Negative	26	2	10		
Positive	1	43	140		
Napsin A				**<0.001**	**<0.001**
Negative	9	40	144		
Positive	18	5	6		
P53				**0.038**	**0.002**
Negative	10	7	103		
Positive	17	38	47		
Ki-67				0.947	**0.005**
lt;50%	8	13	88		
≥ 50%	19	32	62		
LVI				0.116	**0.003**
Absent	20	25	139		
Present	7	20	11		
Distant metastasis				0.783	0.283
Absent	24	39	141		
Present	3	6	9		
Treatment				0.115	0.161
Surgery	2	6	37		
Surgery + Chemotherapy	2	13	28		
Surgery + RT	9	8	40		
Surgery + Chemotherapy + RT	9	18	45		
OS (mouths)				0.155	**0.002**
Average	56	60	63		
Median	56	60	65		

All statistical analyses were performed after elimination of lost follow-up patients.

P1 values are for comparison of CCC and UPSC.

P2 values are for comparison of CCC and EC.

Data in bold means statistically significant.

**Figure 5:**
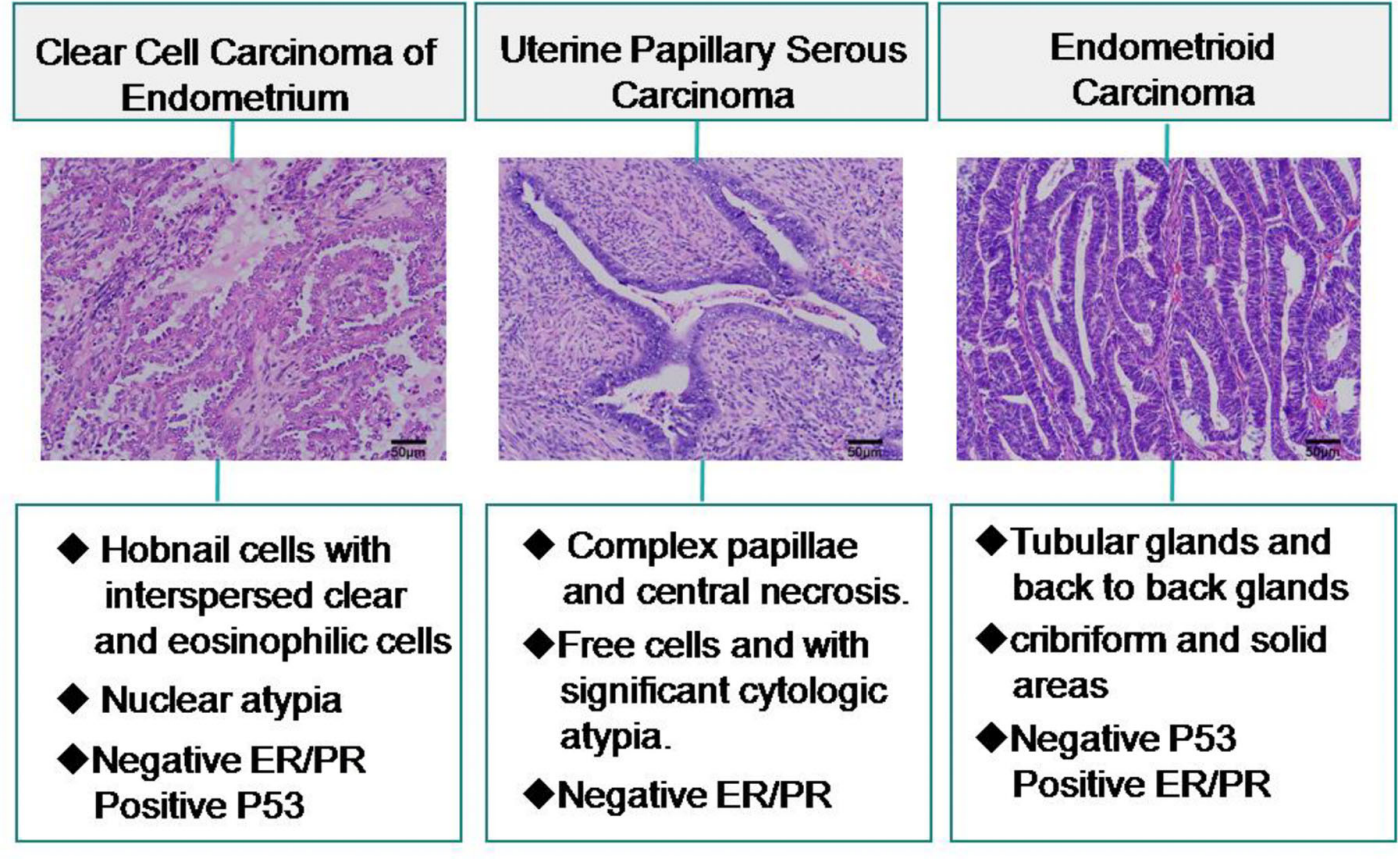
Differential diagnosis of clear cell carcinoma of the endometrium and relevant histopathologic features.

In our cohort, we eliminated lost follow-up patients and selected 150 patients diagnosed with endometrioid carcinoma (EC) with an average age of 56 years (31 - 79 years). 65% (98/150) cases have myometrial invasion inner half involvement. The proportion of ER positive expression was higher 92% (138/150), P53 positive expression was lower 31% (47/150). There were 11 of cases with LVI and 9 of cases with distant metastasis. Patients with an average survival time of 63 months. Compared with CCC, age, treatment, distant metastasis between the two groups had no significance (P > 0.05). There was significance in myometrial invasion, the expression of ER and P53, LVI, and survival time between CCC and EC (P = 0.039, P < 0.001, P = 0.002, P = 0.003, P = 0.002 respectively; **Table 4**). The diagnosis of CCC and its distinction from UPSC and EC is outlined in **Figure 5**

## Discussion

Staging of endometrial cancer follows the surgical and pathological staging adopted by the FIGO, even though it is only based on the depth of myometrial invasion and does not take into account the histological type, tumor grade, tumor size, and other relevant factors affecting prognosis. In recent years, new perspectives based on histology, immunohistochemistry, molecular genetics, prognosis-related factors, and the effect of different management practices on prognosis have emerged. In 2018, Maheshwari performed surgical/pathological staging of endometrial cancer based on important prognosis-related factors (12). He proposed that type II non-endometrioid carcinomas, including clear cell endometrial carcinoma, should be classified as “high level” stage (12). However, to date, there is no consensus on this view.

WHO classifies endometrial cancer into two categories: Type I also known as a classical pathway, occurs under the stimulation of estrogen, with atypical hyperplasia of the endometrium as a precursor, and endometrioid carcinoma is the most common histological type. Type II account for 10%-21% of all endometrial cancers, it is independent of estrogen stimulation, and usually present in older people with endometrial atrophy (13–15). Type II tumors include UPSC, CCC, and undifferentiated carcinoma. CCC is more aggressive and has a worse prognosis compared to other types of endometrial cancer (5, 16, 17). Therefore, our control group comprised UPSC and EC patients. We found that there was no significance in survival time between CCC and UPSC (P > 0.05). Compared to EC, the survival time in CCC was significantly shorter (P = 0.002).

In our cohort, a total of 27 CCC patients performed survival analysis. 14 (52%) of the patients were older than average age at diagnosis (60y), 14 (52%) of the patients were more than average tumor size (3.5cm). 15 (55%) of the patients were MI > 1/2. Older age, big tumor size, deep MI significantly associated with poor prognosis (P = 0.048, P = 0.016, and P = 0.043 respectively). The OS was significantly shorter for CCC patients with stage III and IV than for those with stage I and II (P < 0.001). 7 (26%) of the patients had LVI, and 3 (11.1%) of the patients had distant metastasis. LVI (P = 0.001) and distant metastasis (P < 0.001) were prognostic factors for the OS of CCC patients. 3 (11.1%) patients developed tumor recurrence and the most common metastatic site was liver. These results suggest that CCC have invasive potential.

In this study, the typical clinical presentation of patients with CCC was postmenopausal bleeding before diagnosis. The diagnosis of CCC can be achieved through clinical manifestation, preoperative diagnostic curettage, and endometrial biopsy, as well as in other types of endometrial carcinoma. Endometrial biopsy is a sensitive and accurate method used to evaluate abnormal bleeding (18). Pap smear is not a reliable diagnostic method for endometrioid carcinoma, but it appears that diagnosis can be made following an abnormal smear in CCC patients (19). However, the final diagnosis should rely on examination of histopathological sections. Delaying the diagnosis can have serious consequences, and the 5-year survival rate significantly decreases as the disease progresses (20).

Transvaginal ultrasonography is the first choice for patients with abnormal postmenopausal bleeding (21). Preoperative imaging examination is crucial for evaluating the depth of myometrial invasion and the presence of adnexal and distant metastasis. Magnetic resonance imaging (MRI) and positron emission tomography (PET)/computer tomography (CT) are used in advanced-stage patients with lymphovascular invasion and distant metastasis.

Taking into consideration the rarity of CCC, diagnosis remains a challenge. In this study, the immunohistochemical performance for Napsin A was high, low for p53, a high Ki-67 index, and absence or focal nuclear expression of ER and PR. This is one of the reasons why hormone therapy is not routinely used for CCC treatment (22). Napsin A was higher frequent expression in CCC (67% – 93%) and lower frequent expression in UPSC (8% – 22%) and EC (0 –10%) (23, 24). In EC, ER/PR is positive, while in CCC ER and PR showed negative expression or focal nuclear expression. In UPSC, P53 has a diffuse immunoreactivity, while in CCC P53 showed weakly or moderately positive expression (22, 25). The immunohistochemical features is consistent with our research. These studies contribute to a deeper understanding to distinguish CCC from other subtypes of endometrial cancer.

A previous study suggested that high Ki-67 indices were related to increased tumor proliferation, poor prognosis (26), and decreased survival time. Positive expression of P53 is associated with unfavorable outcomes in CCC (22), and this was confirmed in the present research. The expression of E-cadherin in CCC is significantly lower than that in endometrioid carcinoma, which demonstrates that reduced cohesion of tumor cells is responsible for the more aggressive behavior of CCC (27). Napsin A is located in the cytoplasm and unrelated to patient age, pathological subtype, FIGO stage, degree of infiltration, lymphovascular invasion and OS (28). In our research, the OS was shorter for CCC patients with high Ki-67 index than for those with low Ki-67 index (P = 0.026), positive expression of P53 significantly associated with poor prognosis (P = 0.007), and Napsin A positive expression was unrelated to prognosis (P = 0.119), which is consistent with previous studies.

In the 2013, the Global Cancer Genome (TCGA) study, divided endometrial cancer into four types based on histomorphology and molecular genetics, including polar hyper-mutation, microsatellite instability, low copy number, and high copy number types (29). TCGA emphasizes the importance of classification in prognosis to provide better-individualized treatment. To develop more efficacious molecular targeted therapies, there is an urgent need to determine the molecular characteristics of endometrial cancer.

The molecular features of CCC were not been analyzed by TCGA. Therefore, the molecular characteristics of CCC remain not clearly explored compared to EC and UPSC. Therefore, we performed the immunohistochemical analysis of mismatch repair (MMR) proteins (MSH2, MSH6), P53, PTEN and HER-2 to understand CCC from the perspective of molecular genetics.

Based on molecular profile analysis, the most frequently mutated gene is TP53, followed by KRAS and PIk3CA (30–32). However, in the majority of uterine clear cell carcinoma, there is an absence of mutations in the P53 (13). Positive P53 immunohistochemical staining can indicate P53 gene mutation. Soyoun et al. have reported that P53-mutated was observed in to 18 cases (35%) and P53 wild-type was observed in to 28 cases (54%) (33). Deborah et al. have reported that 11 (34%) cases displayed P53-mutated (31). In this study, approximately 2/3 of CCC patients showed a mutated P53 immunostaining pattern and P53-mutated cases had a shorter survival time compared to P53 wild-type cases.

HER2/neu, also named asc-erbB2. HyoSookBae et al. have reported that overexpression of HER2/neu was observed in only to 2 cases (12.5%), but the amplification of HER2/neu gene was not detected by situ hybridization (FISH) in all 16 CCC cases (10). They also discovered that PTEN loss was seen in 81.3% of 16 CCC cases but the LOH of PTEN was only 6.3% (10). Lien et al. reported that PTEN mutations were not detected in 14 CCC cases (34). 6 (19%) cases showed abnormal expression of MMR proteins and 11% ERBB2 amplifications was detected in 32 CCC cases (31). Also have immunohistochemistry analysis revealed 15 (33.3%) cases loss of MMR proteins in 45 CCC cases (4). In immunohistochemical staining, overexpression of HER2/neu was not found in of our cases, negative expression was found in all 27 cases in our study. Loss of MMR proteins (MSH2 and MSH6) were observed in 7 cases (25.9%). 12 (44.4%) cases showed PTEN loss. MSH2, MSH6 and PTEN have no statistical significance with OS (P = 0.472, P = 0.524 and P = 0.472, respectively) in our study.

Currently, total abdominal hysterectomy and bilateral salpingo-oophorectomy have been established as first-line treatment. Systematic pelvic and para-aortic lymphadenectomy are reported to improve patients prognoses (35). Pre-and/or postoperative chemotherapy and/or radiation have been widely employed. Patients diagnosed with CCC are administered with adriamycin, cisplatinum, and paclitaxel either in a double or triple combination. The triple combination, however, is demonstrated to cause neurologic and hematologic toxicities (1, 36). Radiation therapy is primarily administered in the postoperative adjuvant setting (37), depending on the risk of recurrence and patient-related factors. To explore the relationship between adjuvant therapy and prognosis, a multi-institutional retrospective study reported that brachytherapy was beneficial for survival in stages I – II patients, while chemotherapy was significant for stage III patients (38). On the contrary, Abdulfatah reported that adjuvant radiotherapy alone had a significant impact on a patient’s prognosis (P = 0.012), while the prognosis of patients receiving chemotherapy alone or combined with radiotherapy showed no significant improvement on OS (P=0.202, P=0.229, respectively) (39). External beam radiation (EBRT) or vaginal brachytherapy (VBT) can reduce vaginal stump recurrence, and EBRT is recommended for patients who are not eligible for chemotherapy (40). Some institutional studies have shown that both adjuvant chemotherapy and radiotherapy are found to be beneficial in CCC and UPSC patients (41, 42), while other studies found that the patients treated with chemotherapy had no added benefit (43, 44). There are no universally accepted guidelines for patient’s management (39).

There remain limitations in this article. Firstly, our results show that any adjuvant therapy cannot effectively improve the prognosis of CCC. It is great possibility that this is the single agency study with small sample size, and has the limitation of data accessibility. Secondly, the radiotherapy or chemotherapy regimens for patients of CCC were different. We did not collect the specific radiotherapy regimens and not clear about whether pelvic radiotherapy or vaginal brachytherapy is related to the prognosis of patients. Similarly, patients of CCC receive different chemotherapy cycles. We have made an immunohistochemical analysis of CCC instead of whole exome sequencing, which cannot identify the degree of genes mutation. In our cohort, there was no significant difference in survival outcomes between UPSC and CCC. Therefore, further stratified analyses with larger populations by combination with other medical units are required.

Considering the rarity of CCC, prospective retrospective studies are difficult to perform. Therefore, this retrospective study is valuable, as a representative sample of Eastern China, to make up for deficiencies in clinical parameters, pathological variables, immunohistochemical characteristics and survival data. We discussed 10 markers and evaluated molecular characteristics of CCC. PTEN, MSH2, MSH6 and HER-2 are not specific and sensitive antibodies for detecting CCC, and have no statistical significance with prognosis. This study demonstrates that age at diagnosis, FIGO stage, MI, tumor size, high Ki-67 index, positive expression of P53, lymphovascular invasion and distant metastasis are significantly associated with OS.

## Data Availability Statement

The original contributions presented in the study are included in the article/supplementary material. Further inquiries can be directed to the corresponding authors.

## Ethics Statement

The studies involving human participants were reviewed and approved by First Affiliated Hospital of Bengbu Medical University. The patients/participants provided their written informed consent to participate in this study. Written informed consent was obtained from the individual(s) for the publication of any potentially identifiable images or data included in this article.

## Author Contributions

DC studied concept and design; ZZ, ZB, PG, LZ acquired data. ZZ, ZB and JY analyzed data. ZZ drafted the manuscript. TL revises the manuscript. ZZ, PG provided acquisition, analysis and interpretation of data, and statistical analysis. All authors read and approved the final manuscript.

## Funding

This work was supported in part by the Nature Science Major and Key Program of College and University of Anhui Province (No.KJ2020A0559), the Natural Science Research Program of Education Bureau of Anhui Province (NO. gxyq2017032), the Nature Science Key Program of College and University of Anhui Province (NO.KJ2016A460).

## Conflict of Interest

The authors declare that the research was conducted in the absence of any commercial or financial relationships that could be construed as a potential conflict of interest.

## Publisher’s Note

All claims expressed in this article are solely those of the authors and do not necessarily represent those of their affiliated organizations, or those of the publisher, the editors and the reviewers. Any product that may be evaluated in this article, or claim that may be made by its manufacturer, is not guaranteed or endorsed by the publisher.
